# Supplementary material for: 3D motion tracking display enabled by magneto-interactive electroluminescence
Source: Nat Commun. 2020 Nov 27;11:6072. doi: 10.1038/s41467-020-19523-0 (PMC7695719; doi:10.1038/s41467-020-19523-0)
Supplement: Supplementary file 1 — Supplementary Information [file 41467_2020_19523_MOESM1_ESM.docx]

**Supplementary Information (SI)**

*3D Motion Tracking Display Enabled by Magneto-interactive Electroluminescence*

Seung Won Lee^1,4^, Soyeon Baek^1,4^, Sung-Won Park^2^, Min Koo^1^, Eui Hyuk Kim^1^, Seokyeong Lee^1^, Wookyeong Jin^1^, Hansol Kang^1^, Chanho Park^1^, Gwangmook Kim^1^, Heechang Shin^2^, Wooyoung Shim^1^, Sunggu Yang^3^, Jong-Hyun Ahn^2^ & Cheolmin Park^1^*

^1^Department of Materials Science and Engineering, Yonsei University, Seoul 120-749, Korea.

^2^Department of Electrical and Electronic Engineering, Yonsei University, Seoul 120-749, Korea.

^3^Department of Nano-Bioengineering, Incheon National University, Incheon 22012, Korea

^4^These authors contributed equally: Seung Won Lee, Soyeon Baek

**Supplementary Figures 1-30**

**Supplementary Tables 1**


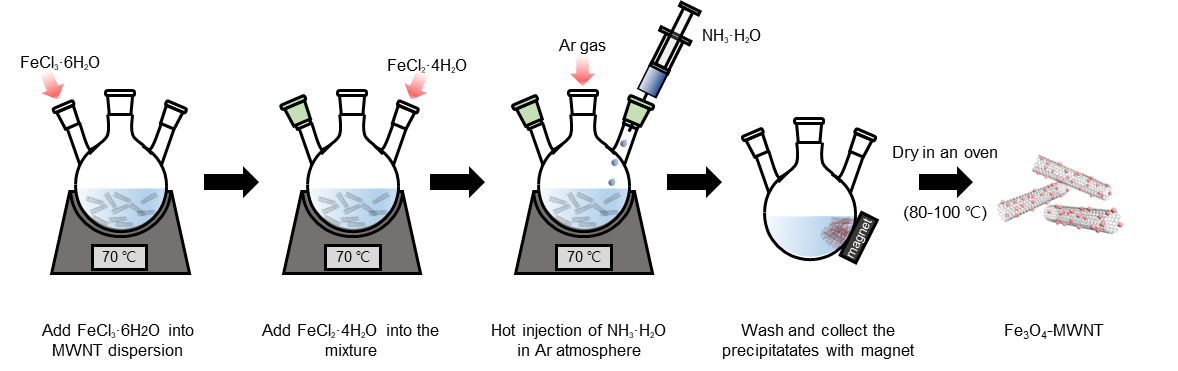


**Supplementary Figure 1.** Synthesis processes of iron-oxide nanoparticles decorated with multi-walled carbon nanotubes (Fe_3_O_4_-MWNTs) for the magnetoactive fluid.

**
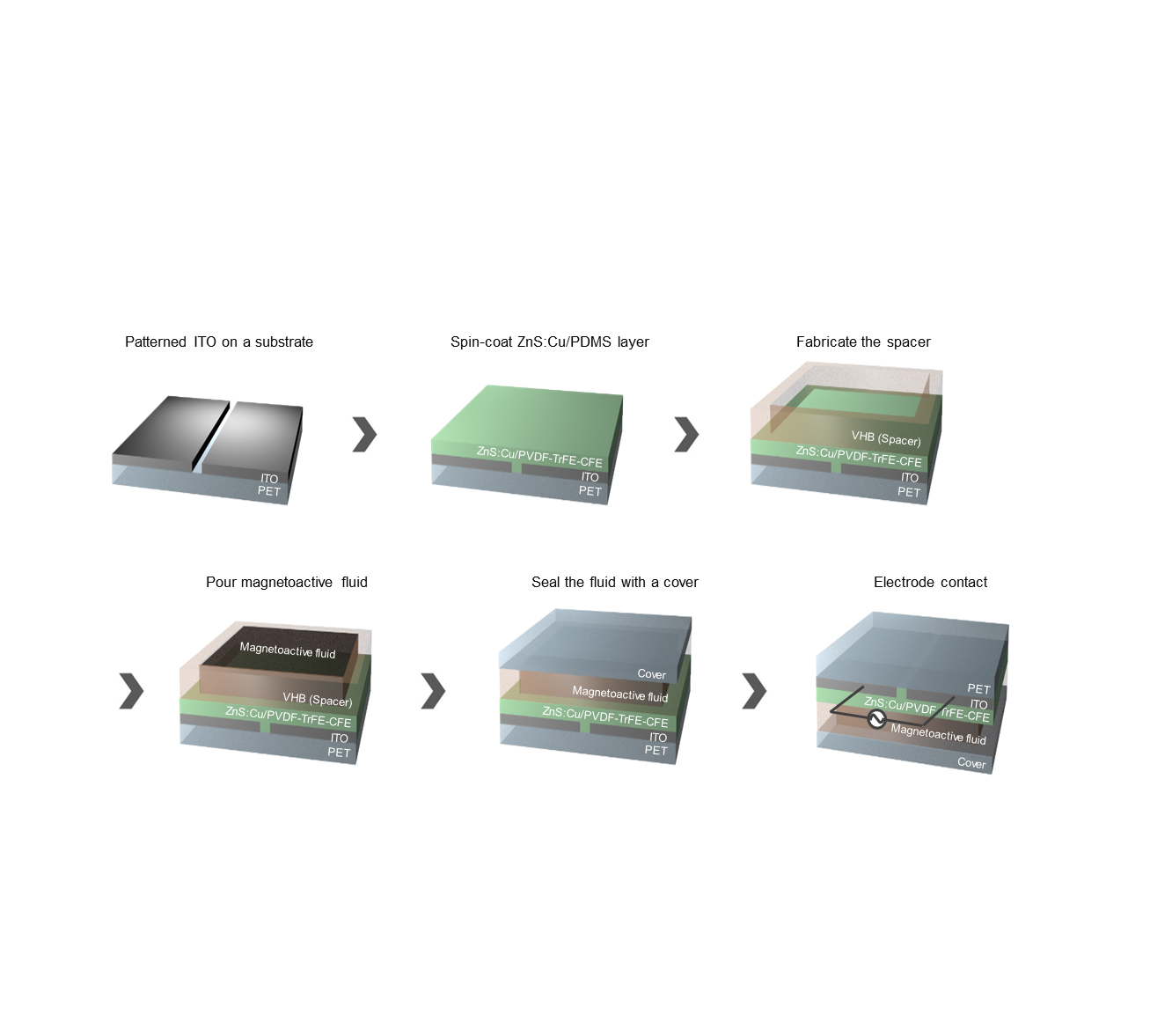
**

**Supplementary Figure 2.** Fabrication process of an NV-MED.


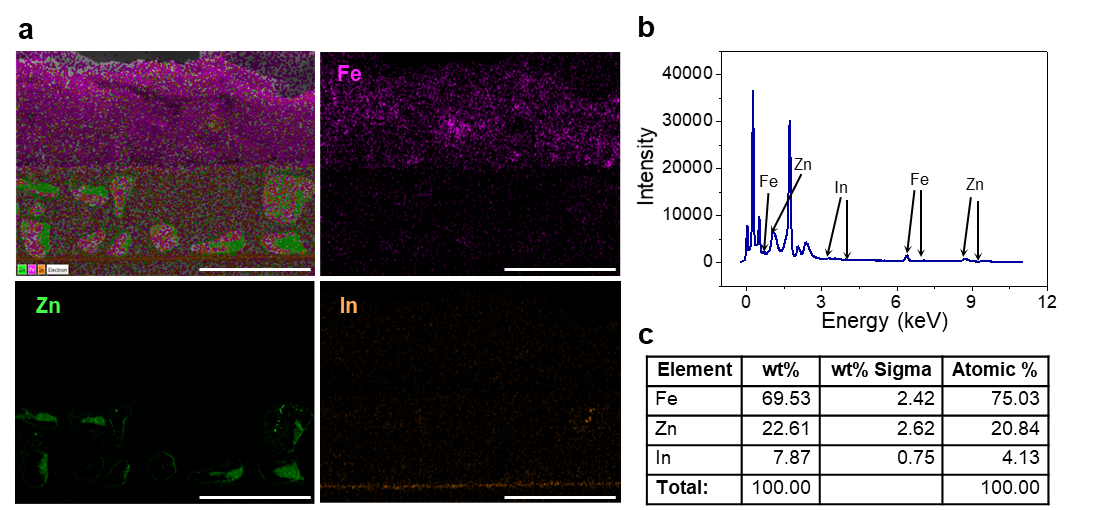


**Supplementary Figure 3.** Chemical analysis of the constituent layers of an NV-MED. **a**. Two-dimensional mapping of a cross section of an NV-MED. The images show the maps for Fe (magenta), Zn (green), and In (orange) (scale bars: 50 μm). **b**. Energy spectrum and **c**. energy-dispersive spectroscopy (EDS) elemental analysis of SEM-energy-dispersive X-ray (EDX) measurement.

**Supplementary Note 1:**

**Operation mechanism of an NV-MED**

The NV-MED with a well-dispersed magnetoactive fluid was exposed to a magnetic field. The percolation network of Fe_3_O_4_-MWNTs programmed using magnetic field, with a certain network density placed on the emissive layer (Supplementary Fig. 4a), acts as a magnetoactive conductive bridge layer (write step of Supplementary Fig. 4b). The magnetic field information (f_1_) programmed in the percolation network density was read under an AC field in terms of visible light coming from the emissive layer after the field was removed (read step of Supplementary Fig. 4b). The programmed percolation network was erased by applying an opposite magnetic field sufficient enough to remove the networked conductive bridge near the emissive layer, making the Fe_3_O_4_-MWNTs in NV-MED randomised in n-hexadecane, as shown in the erase step of Supplementary Fig. 1b. After removing the conductive bridge layer in the NV-MED, another magnetic field (f_2_) was applied, followed by the writing of a newly programmed percolation network in the NV-MED. This rewrite process developed a conductive network dependent upon the intensity of the magnetic field. The EL corresponding to the percolated network arising from magnetic field (f_2_) was characterised in the AC-based read process again, yielding a novel non-volatile, rewritable magnetic field sensing memory display. It should be noted that unlike the single-domain superparamagnetic iron-oxide particles of ~10 nm in diameter, the percolated network of Fe_3_O_4_-MWNTs acts as a multi-domain iron-oxide cluster. Therefore, the network should have a weak residual magnetism and retain its shape without re-dispersion by Brownian motion after the disappearance of the magnetic field, giving rise to the excellent retention properties of the NV-MED.


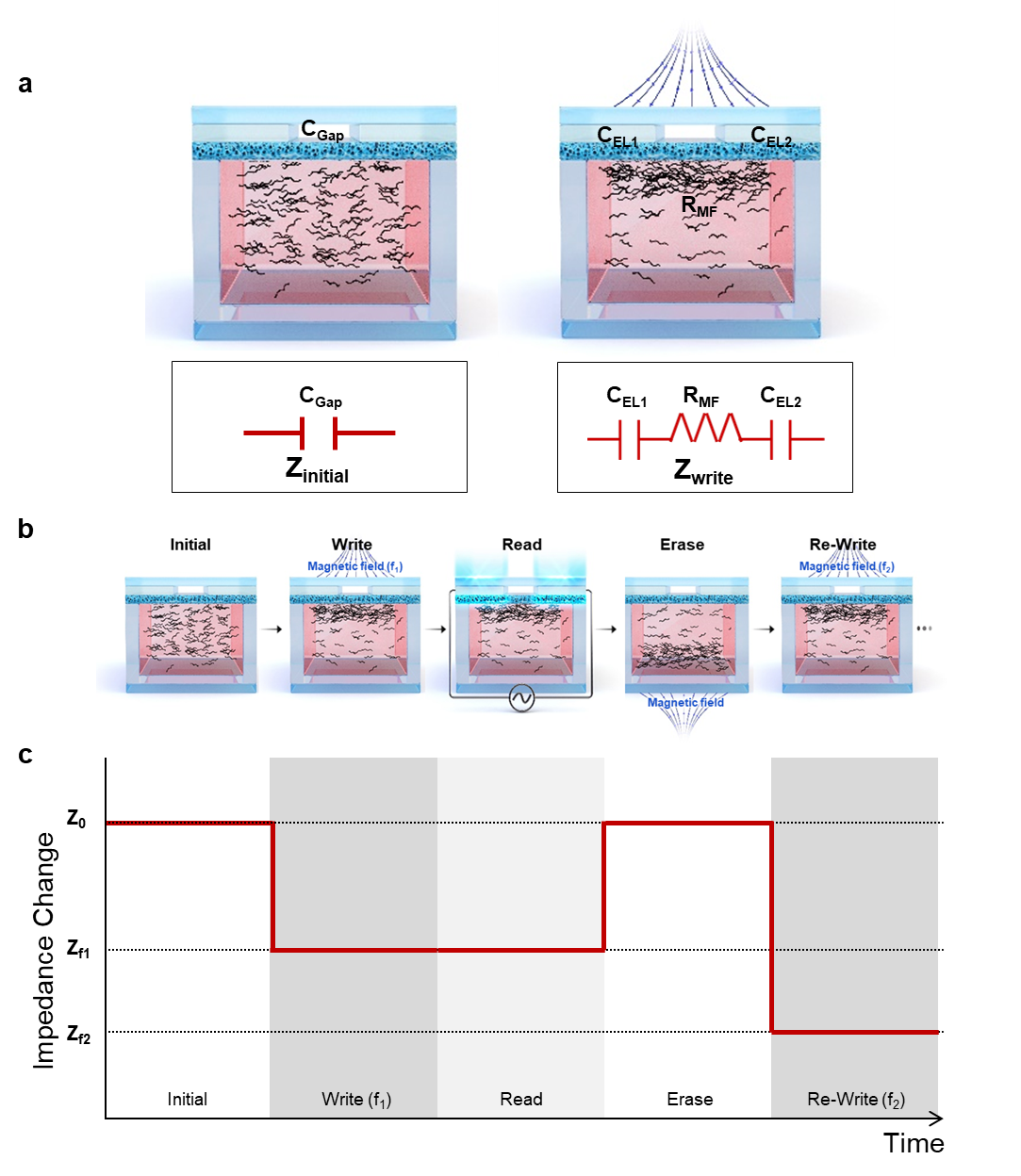


**Supplementary Figure 4.** Operation mechanism of an NV-MED. **a**. Equivalent circuits corresponding to the state of an NV-MED with and without a magnetic field. **b**. Schematic of the non-volatile writing, reading, and erasing of the information of the magnetic field with an NV-MED. **c**. Schematic of the impedance change in the writing, reading, and erasing process under the magnetic field.

**Supplementary Note 2:**

**Characterisation of magnetoactive Fe_3_O_4_-MWNTs** **conducting fluid**

Before investigating magnetic field sensing, visualisation, and memorisation performance of our NV-MED, we confirmed the physical and chemical properties of the synthesised Fe_3_O_4_-MWNTs and further examined the effects of magnetoactive fluid layer on the sensing and light-emitting performance of an NV-MED using the ratio of Fe_3_O_4_ to MWNTs and n-hexadecane content. The typical morphology of as-synthesised Fe_3_O_4_-MWNTs was obtained using SEM, as shown in Supplementary Fig. 5a. Representative transmission electron microscopy (TEM) images for an Fe_3_O_4_-MWNT shown in Supplementary Fig. 5b suggest that Fe_3_O_4_ particles of ~10–20 nm in diameter are randomly decorated on the MWNT surface. The high-angle annular dark field (HAADF) scanning TEM (STEM) image and related energy-dispersive X-ray spectrum (EDX) of a small fragment from the Fe_3_O_4_-MWNT present a homogenous distribution of elements, as shown in Supplementary Fig. 5c.

The Raman spectra of MWNTs, Fe_3_O_4_ nanoparticles, and Fe_3_O_4_-MWNTs are shown in Supplementary Fig. 6. The G band (1580.5 cm^-1^) reflects the purity and regular structure of the MWNT, whereas the D band (1346.2 cm^-1^) corresponds to the defects at the surface of the MWNT. The peak centred at 2699.2 cm^-1^ is assigned to the D* band of the MWNT. Some extra peaks in Fe_3_O_4_-MWNTs at lower wavenumbers, including 274.8, 481.6, and 588.7 cm^-1^, are ascribed to the Fe–O and Fe–C bonds, confirming that Fe_3_O_4_ nanoparticles are successfully anchored onto the surface of MWNTs.

X-ray powder diffraction (XRD) results for Fe_3_O_4_-MWNTs clearly show the characteristic graphitic peak centred at 2θ = 26.5°, which indicates that the MWNTs are well preserved during the synthesis of the Fe_3_O_4_-MWNTs, as shown in Supplementary Fig. 7. According to the MDI Jade database (JCPDS No. 89-0691) for Fe_3_O_4_, the characteristic diffraction peaks centred at 2θ = 30.2°, 35.7°, 43.3°, 53.8°, 57.3°, and 62.9° are associated with the (220), (311), (400), (422), (511), and (440) planes, respectively.

The magnetisation behaviour of the Fe_3_O_4_-MWNTs with different volume ratios of MWNT:Fe_3_O_4_ were examined as a function of magnetic field, and the results are shown in Supplementary Fig. 8. Saturation magnetisations of ~30 emu/g were obtained in the Fe_3_O_4_-MWNTs (MWNT:Fe_3_O_4_ = 1:1.5) and in those with higher Fe_3_O_4_ contents.


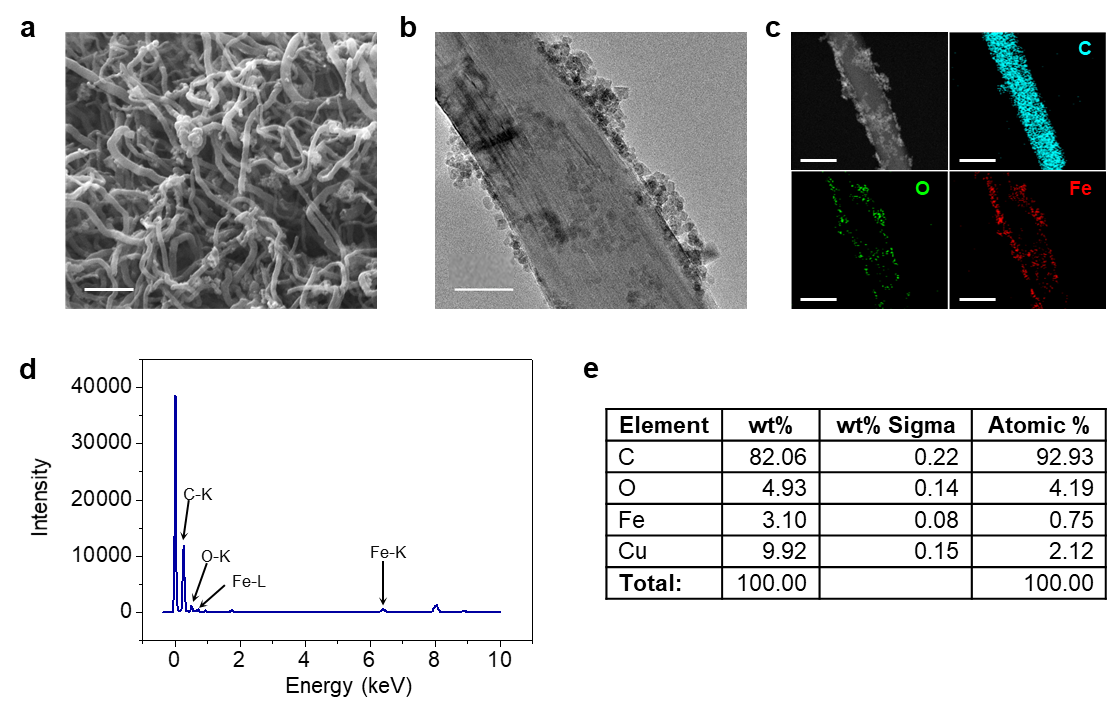


**Supplementary Figure 5.** Scanning TEM (STEM) EDS spectrum and elemental analysis of Fe_3_O_4_-MWNTs. **a**. SEM image of the typical morphology of as-synthesised Fe_3_O_4_-MWNTs (scale bar: 500 nm). **b**. Representative TEM images for a Fe_3_O_4_-MWNT (scale bar: 50 nm). **c**. The high-angle annular dark field image and EDX spectrum of a small fragment from the Fe_3_O_4_-MWNTs. The images show the maps for C (blue), O (green), and Fe (red) (scale bar: 100 nm). **d**. EDS spectrum of C–K, O–K, Fe–L, and Fe–K. **e**. EDS elemental analysis of C, O, and Fe.


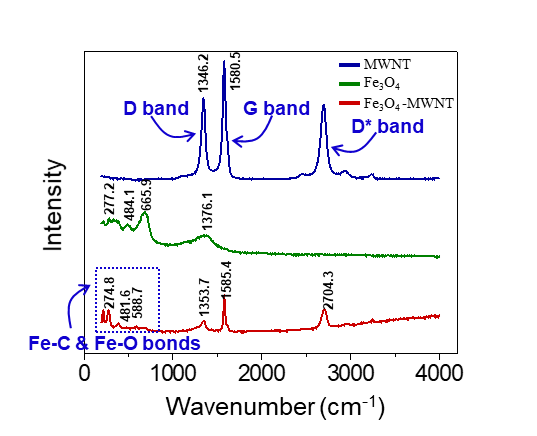


**Supplementary Figure 6.** The Raman spectra of MWNTs, Fe_3_O_4_ nanoparticles, and Fe_3_O_4_-MWNTs.


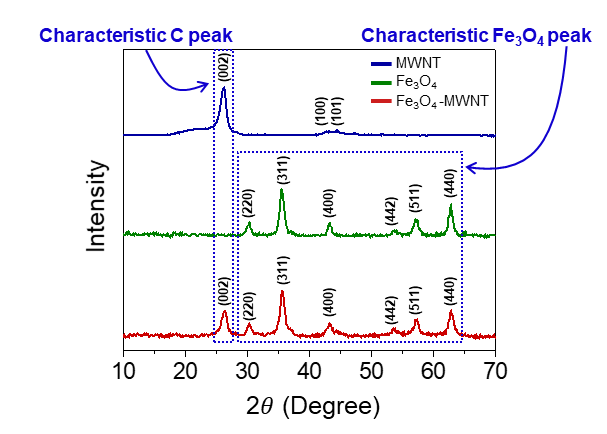


**Supplementary Figure 7.** XRD pattern of Fe_3_O_4_-MWNTs.


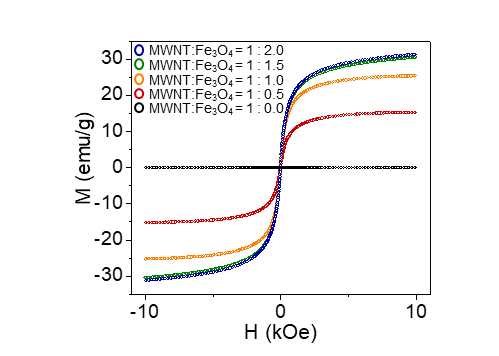


**Supplementary Figure 8.** Magnetisation of Fe_3_O_4_-MWNTs as a function of magnetic field. The specific saturation magnetisations of ~30 emu/g were obtained in Fe_3_O_4_-MWNTs (MWNT:Fe_3_O_4_ = 1:1.5). The results are lower than those for bulk magnetite and also indicate that the variation in composition affected the susceptibility values.

**Supplementary Note 3:**

**Optimisation of Fe_3_O_4_-MWNTs** **magnetoactive conducting fluid in an NV-MED**

We also examined the impedance of the percolated network channel of Fe_3_O_4_-MWNTs with different volume ratios of MWNT:Fe_3_O_4_ as a function of the imposed magnetic field. The impedance of a channel developed near two ITO parallel electrodes in a magnetic field was measured under an AC field, as schematically shown in Supplementary Fig. 9a. The channel impedance decreases with magnetic field owing to the formation of the percolated network of Fe_3_O_4_-MWNTs. When the volume ratio of Fe_3_O_4_ to MWNT increases, the impedance drop with magnetic field was more abrupt, and no significant difference was observed when the volume ratio of Fe_3_O_4_ was greater than 1.5, as shown in Supplementary Fig. 9b. A plot of the impedance variation in Supplementary Fig. 9c as a function of magnetic field based on Supplementary Fig. 9b clearly shows that Fe_3_O_4_-MWNTs with an Fe_3_O_4_ volume ratio greater than 1.5 gives rise to the largest impedance change, which will be beneficial for the sensing of many magnetic field levels with distinct impedance margins between the two neighbouring magnetic fields. For these reasons, we determined to use Fe_3_O_4_-MWNTs with a 1:1.5 volume ratio to develop our NV-MED.

We also optimised the concentration of Fe_3_O_4_-MWNTs in n-hexadecane and chose a magnetoactive fluid containing 1.8 wt% Fe_3_O_4_-MWNTs because it yielded the best EL performance from the viewpoint of low onset magnetic field and high brightness, without significantly harming the fluid stability, as shown in Supplementary Fig. 10.

With the optimised conditions of Fe_3_O_4_-MWNTs in n-hexadecane, we confirmed the development of the percolated network of Fe_3_O_4_-MWNTs arising from magnet placed on the NV-MED; the results are shown in Supplementary Fig. 11a. A black spot of ~5 mm in diameter was instantly developed, arising from the aggregation of Fe_3_O_4_-MWNTs when a cylinder-shaped magnet of ~5 mm in diameter was placed on the top surface of an NV-MED. The microstructure of the black spot clearly shows the Fe_3_O_4_-MWNTs randomly aggregated, depending on the intensity of the magnetic fields. The SEM images in Supplementary Fig. 11b confirm that the density of the percolated network increases with the applied magnetic fields.


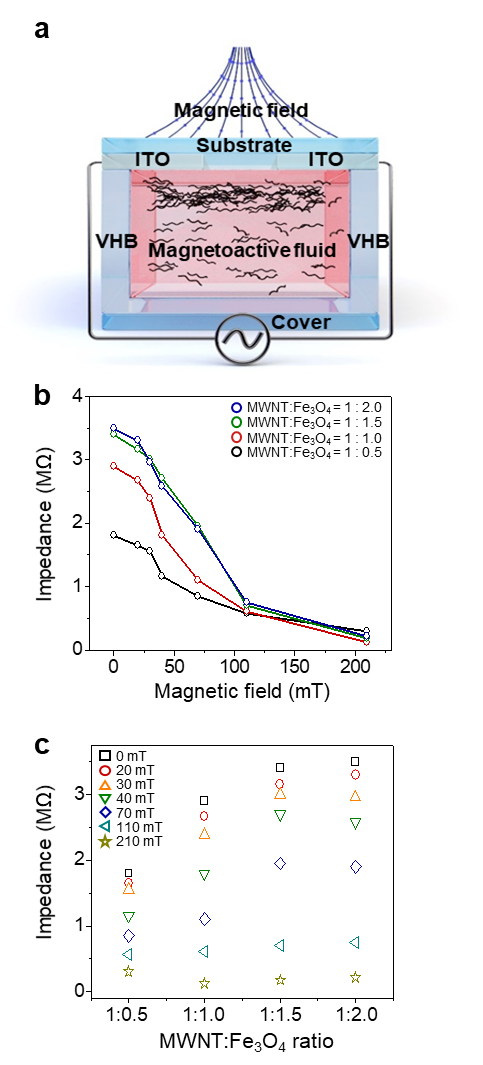


**Supplementary Figure 9.** Impedance of percolated network channel of Fe_3_O_4_-MWNTs. **a**. Schematic of the Fe_3_O_4_-MWNTs channel developed near two ITO parallel electrodes upon the presence of a magnetic field was measured under an AC field. **b**. Plots of the change in impedance (Z) of parallel-type AC devices as a function of magnetic field. The channel impedance decreases with magnetic field owing to the formation of a percolated network of Fe_3_O_4_-MWNTs. **c**. Variation in the impedance of the devices under a different magnetic field as a function of MWNT:Fe_3_O_4_ ratios.


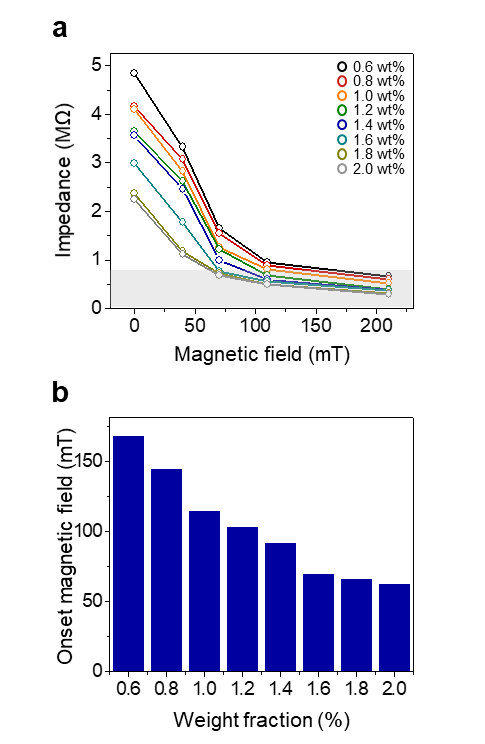


**Supplementary Figure 10.** Threshold resistance and onset magnetic field of an NV-MED for light emission. **a**. Changes in impedance with different weight ratios of Fe_3_O_4_-MWNT to hexadecane as a function of the magnetic field. Various weight ratios from 0.6 to 2.0 wt% were investigated. The impedance range allowing light emission under an AC field is shaded, displaying the variation in the onset magnetic field for light emission with the various ratios. **b**. Onset magnetic field for light emission as a function of weight ratio of Fe_3_O_4_-MWNT to hexadecane. Magnetoactive fluid containing 1.8 wt% Fe_3_O_4_-MWNTs in n-hexadecane was chosen because it gave rise to the optimum EL performance from the viewpoint of low onset magnetic field and high brightness without significantly harming the fluid stability.


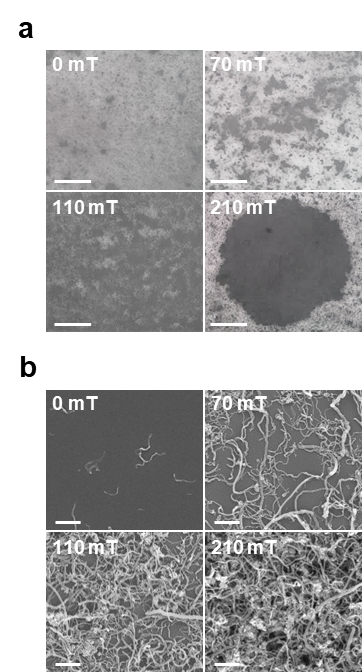


**Supplementary Figure 11.** Morphologies of the magnetoactive percolated networks of Fe_3_O_4_-MWNTs. **a**. Optical microscopic images of the Fe_3_O_4_-MWNTs in the initial state and under various magnetic fields, i.e. 70, 110, and 210 mT were used (scale bars: 100 μm). **b**. SEM images of Fe_3_O_4_-MWNTs in the initial state and under magnetic fields. The microstructure of the black spot clearly shows that the Fe_3_O_4_-MWNTs were randomly aggregated depending on the intensity of magnetic fields (scale bars: 1 μm).
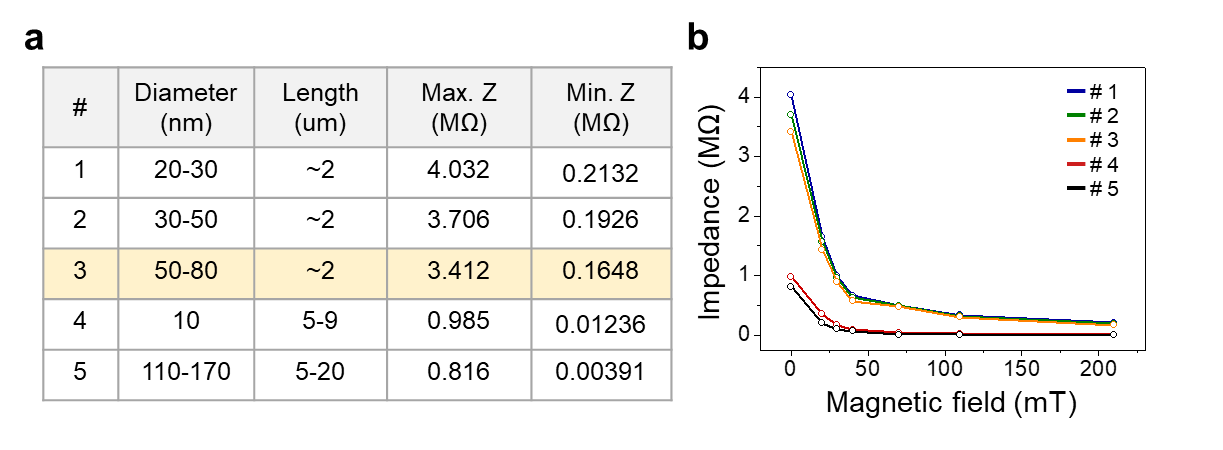


**Supplementary Figure 12.** Electrical characteristics of Fe_3_O_4_-MWNTs with various lengths and diameters of the MWNTs. **a**. Table of impedance characteristics with the MWNTs. **b**. Changes in impedance of the MWNTs in hexadecane as a function of the magnetic field. The diameter of the MWNTs does not significantly affect the impedance characteristics. As the length of the MWNTs increases, the impedance are decreased. The magnetoactive fluid with long MWNTs are less suitable for our NV-MED due to low EL sensitivity and poor fluid stability.


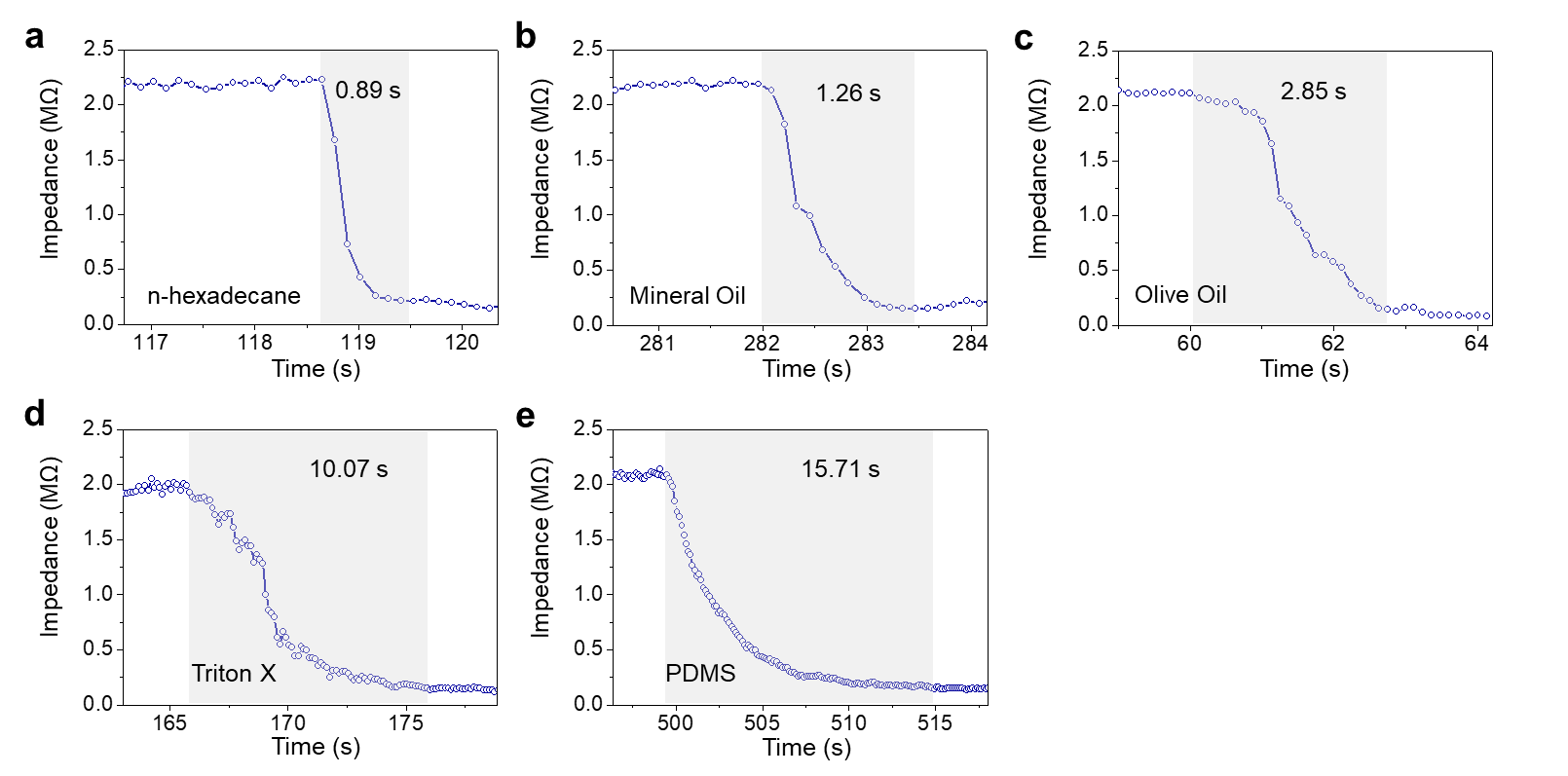


**Supplementary Figure 13.** Electrical characteristics of Fe_3_O_4_-MWNTs in solvents with different viscosities. Changes in impedance of Fe_3_O_4_-MWNTs with various solvents of (**a**) n-hexadecane, (**b**) mineral oil, (**c**) olive oil, (**d**) triton X, (**e**) PDMS under magnetic field (210 mT). The response time of the NV-MED increased with the viscosity of a solvent.


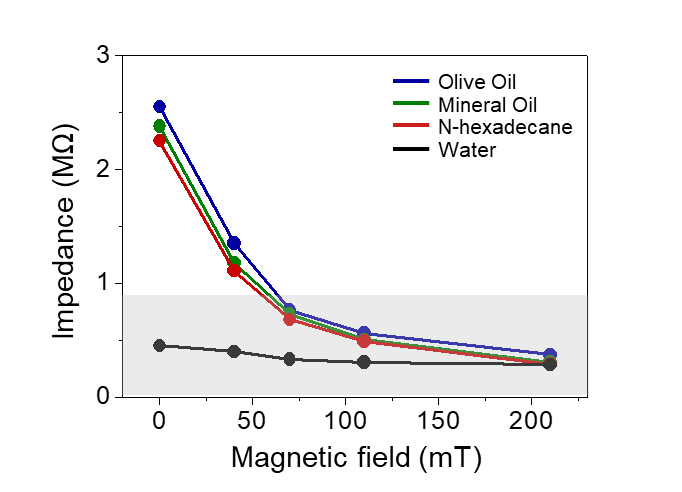


**Supplementary Figure 14.** Electrical characteristics of Fe_3_O_4_-MWNTs in various biocompatible solvents. Non-polar solvent is more suitable for our NV-MED for higher sensitivity to magnetic field.


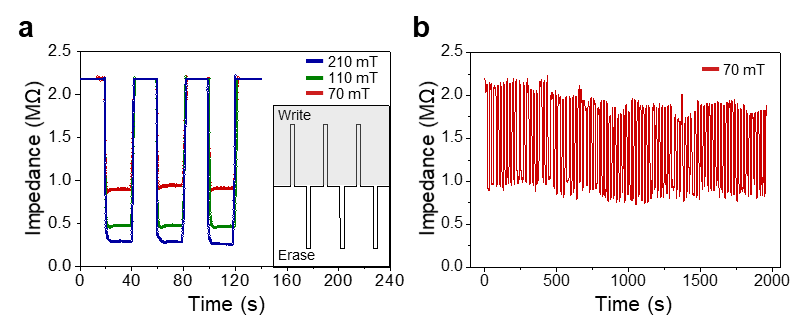


**Supplementary Figure 15.** Impedance properties for sensing and memorisation of the magnetic field in an NV-MED. **a**. Variation in impedance of an NV-MED in writing and erasing with different magnetic fields. The change in the impedance increased with the strength of the magnetic field. The inset shows the response time for the impedance change in the writing and erasing process. **b**. The impedance changes barely changed even after 100 programme/erase cycles with a magnetic field of 210 mT.


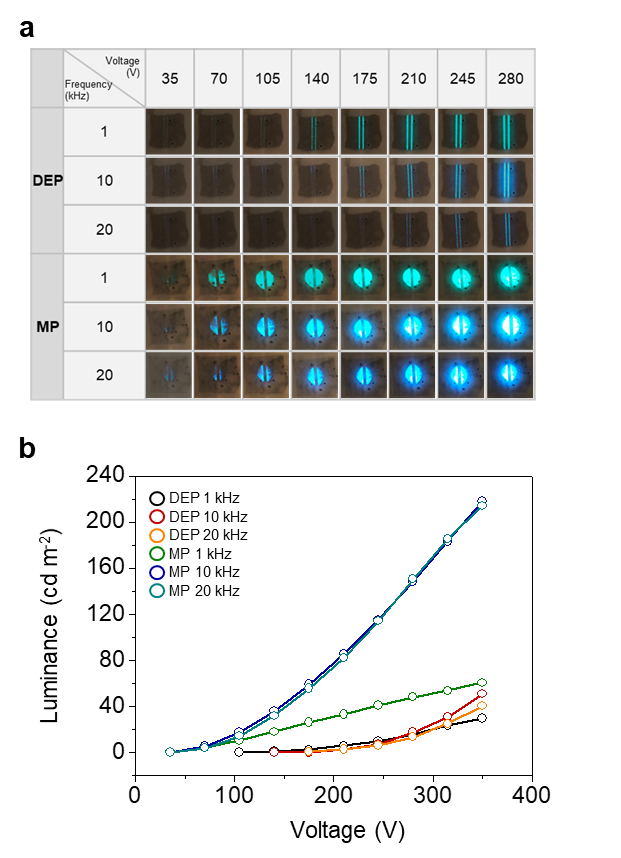


**Supplementary Figure 16.** EL characteristics of an NV-MED arising from electrophoresis and magnetophoresis. **a**. Photographs of an NV-MED by both dielectrophoresis and magnetophoresis under different AC frequencies and voltages (gap size: 1 mm). **b**. Luminance versus voltage (L–V) characteristic of an NV-MED resulting from dielectrophoresis and magnetophoresis. AC frequencies of 1, 10, and 20 kHz were used. In addition, the dielectrophoretic power observed without a magnetic field is orders of magnitude weaker than both the fringe and magnetophoretic fields, making the contribution from the dielectrophoresis negligible in our NV-MED.


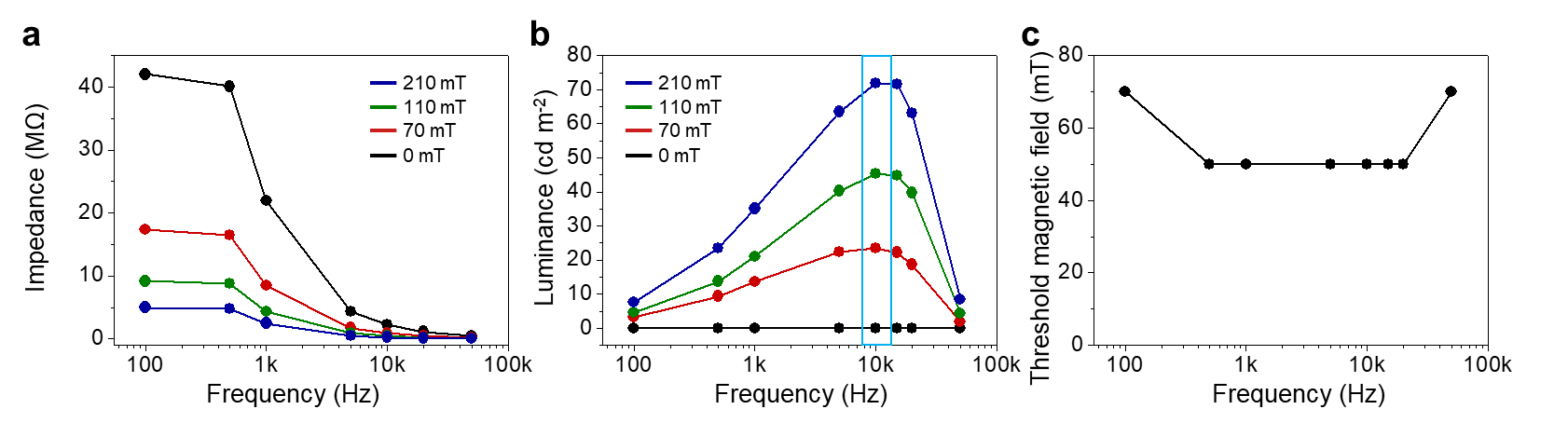


**Supplementary Figure 17.** Impedance and EL characteristics of an NV-MED as a function of frequency. **a**. Impedance of NV-MED as a function of frequency under different applied magnetic fields from 0 to 210 mT. **b**. EL intensity of NV-MED as a function of frequency under different applied magnetic fields from 0 to 210 mT. **c**. Threshold magnetic field of NV-MED as a function of frequency.


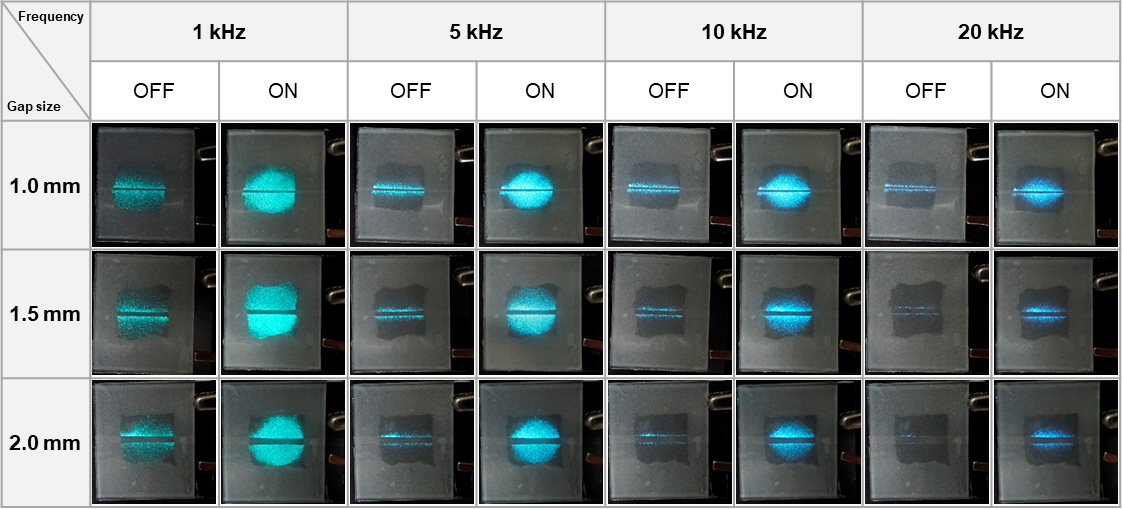


**Supplementary Figure 18.** Photographs of light emission dependent on the gap size of the electrodes. The gap sizes of 1.0, 1.5, and 2.0 and AC frequencies of 1, 5, 10, and 20 were considered.


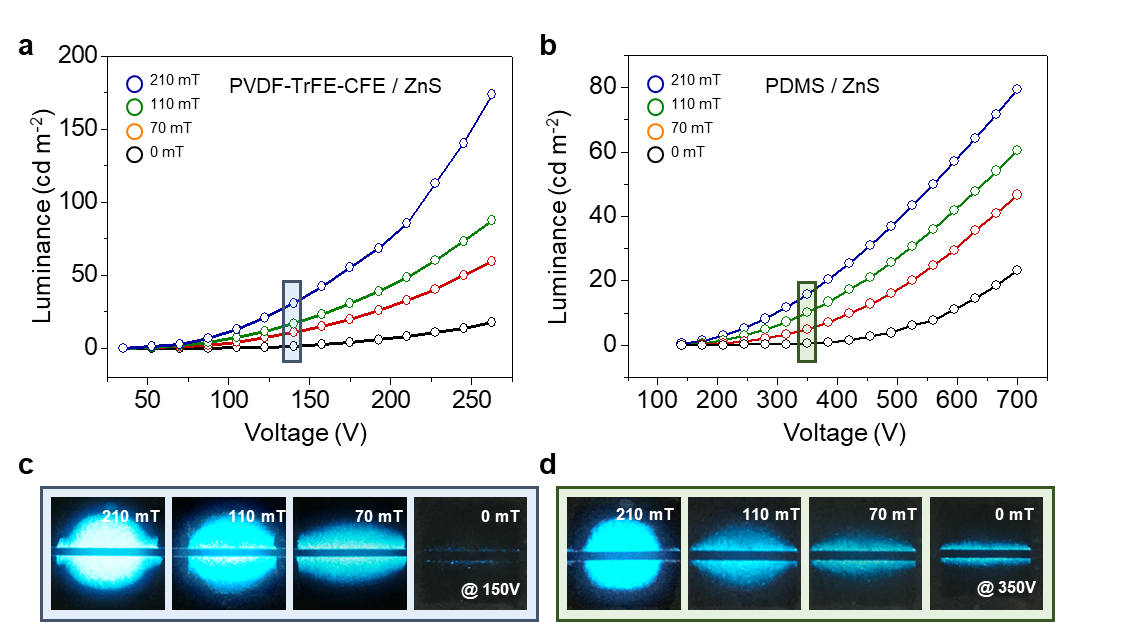


**Supplementary Figure 19.** L–V characteristics of NV-MED devices with different magnetic fields. **a**. L–V characteristics of NV-MED with ZnS:Cu/PVDF-TrFE-CFE composite layer under different magnetic fields. **b**. L–V characteristics of NV-MED with ZnS:Cu/poly(dimethyl siloxane) (PDMS) composite layer under different magnetic fields. **c**. Variation in EL intensity of an NV-MED with ZnS:Cu/PVDF-TrFE-CFE composite layer under different magnetic fields. **d**. Variation in EL intensity of an NV-MED with ZnS:Cu/PDMS composite layer under different magnetic fields. The NV-MED with ZnS:Cu/PVDF-TrFE-CFE operated at the voltage lower than that with PDMS owing to the dielectric constant of PVDF-TrFE-CFE higher than that of PDMS.


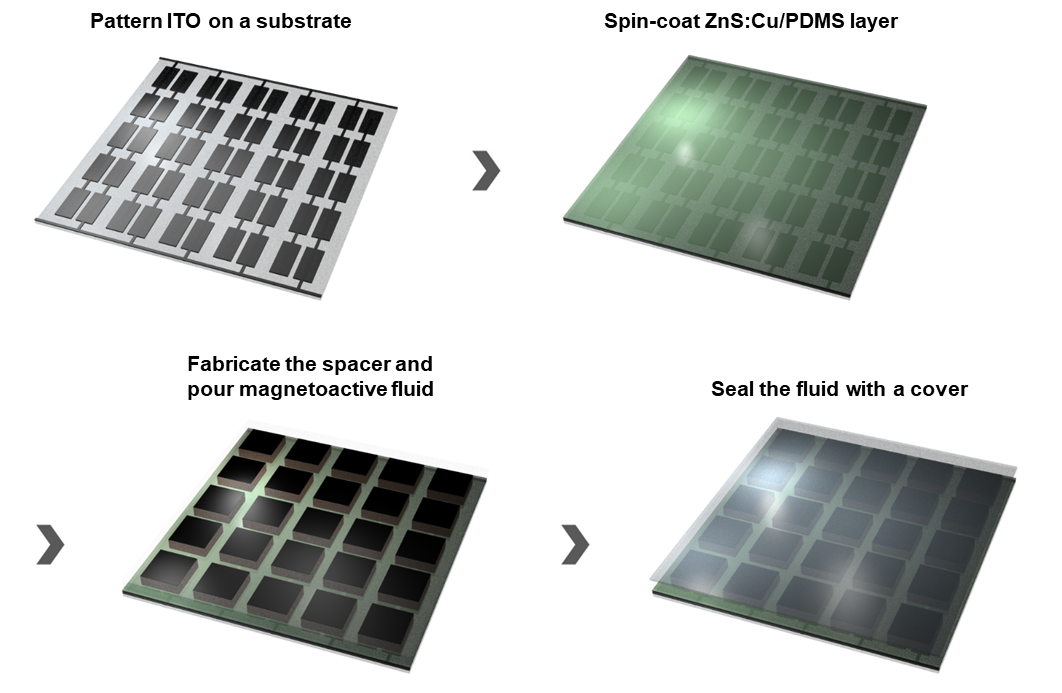


**Supplementary Figure 20.** Fabrication process of an NV-MED array.


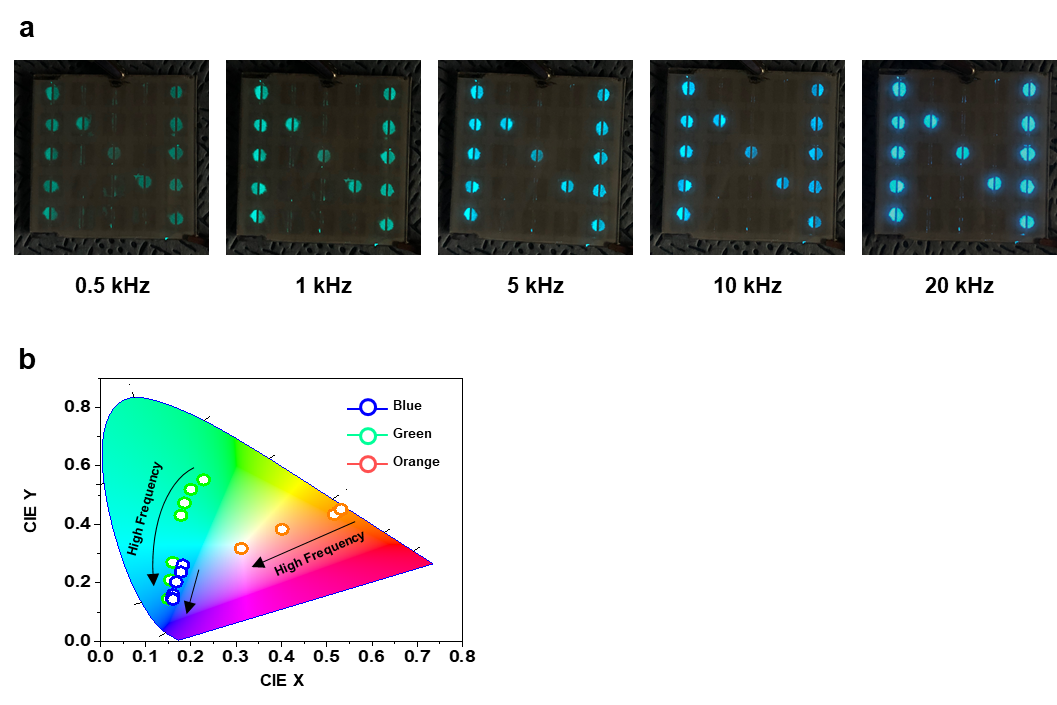


**Supplementary Figure 21.** Colour shift of an NV-MED array. Colour change in an NV-MED array with different AC frequencies from 0.5 to 20 kHz.


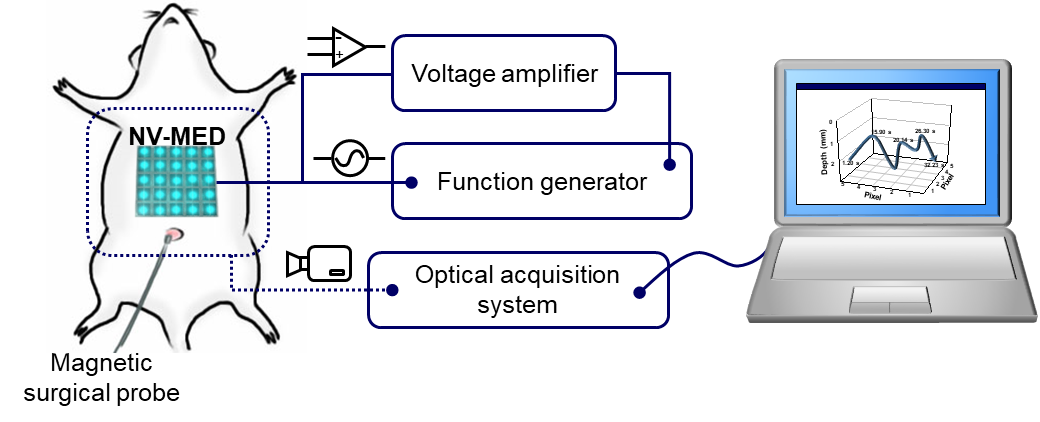


**Supplementary Figure 22.** Schematic of the 3D surgery monitoring and recording system. An optical acquisition system with a camcorder was employed to capture the luminescence induced by a magnetic surgical probe during intraoperation inside the rat. Software was programmed to display the live image as well as the overall trajectory of the luminescence. As the magnetic surgical probe moves to the rat intraperitoneal target organs, such as the liver and stomach, surgical pathways can be electrically and optically stored with the characteristic EL recorded on each pixel with the precise x and y location. It can be explicitly observed that both impedance and luminescence intensity considerably varies along the 3D (x, y, z) trajectory because the applied magnetic field is a function of the depth (z) of the magnetic surgical probe and is directly affected by the response time to the switching ON and OFF of the NV-MED during the operation with constant velocity (Supplementary Fig. 22). After data processing via MatLab, a quantitative 3D monitoring of the luminescence intensity can be performed.


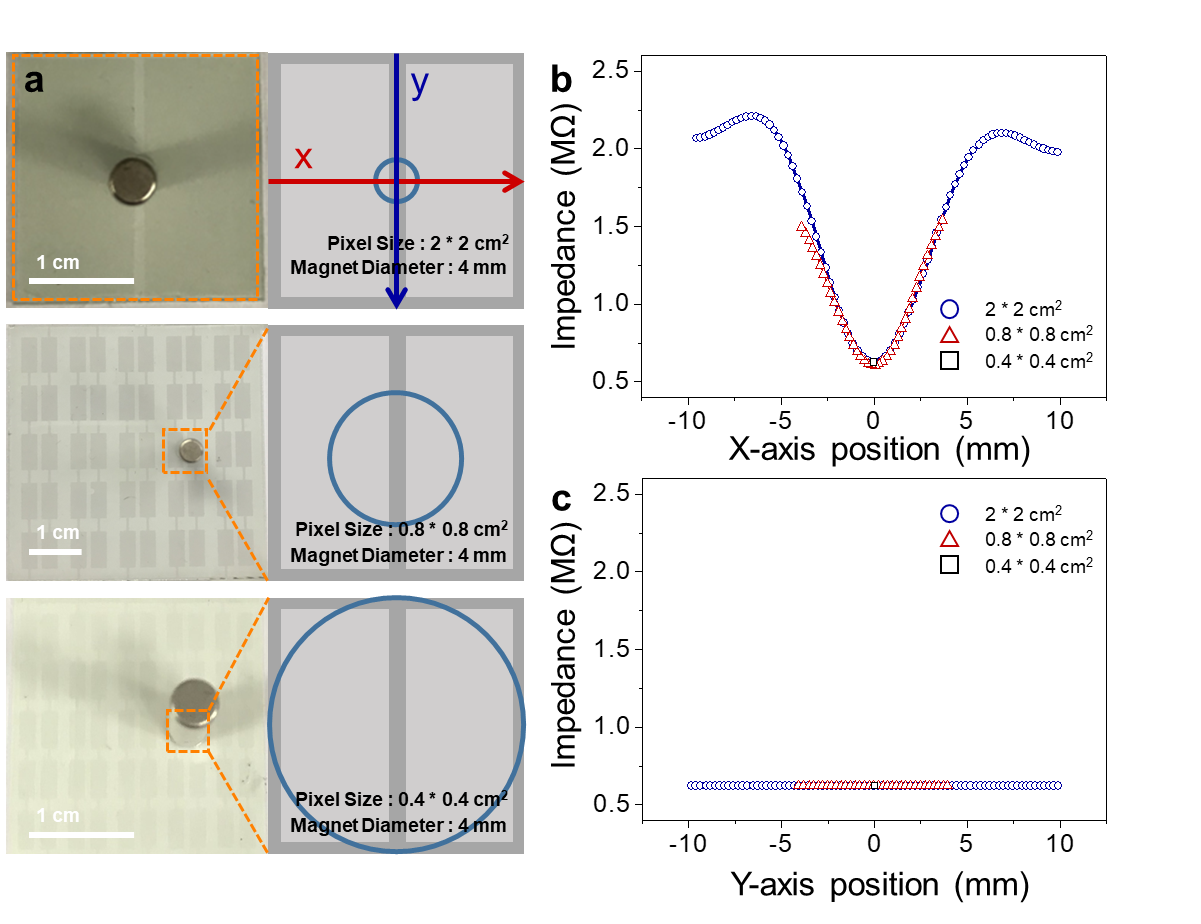


**Supplementary Figure 23.** **a**. Photographs of NV-MED pixel arrays with various pixel widths and magnets with different diameters used in the magnetic surgical probe. Impedance characteristics according to the change of the position of the magnetic probe's (**b**) x and (**c**) y axis in the cases of the NV-MEDs with the pixels of 20, 8, 4 mm in width.


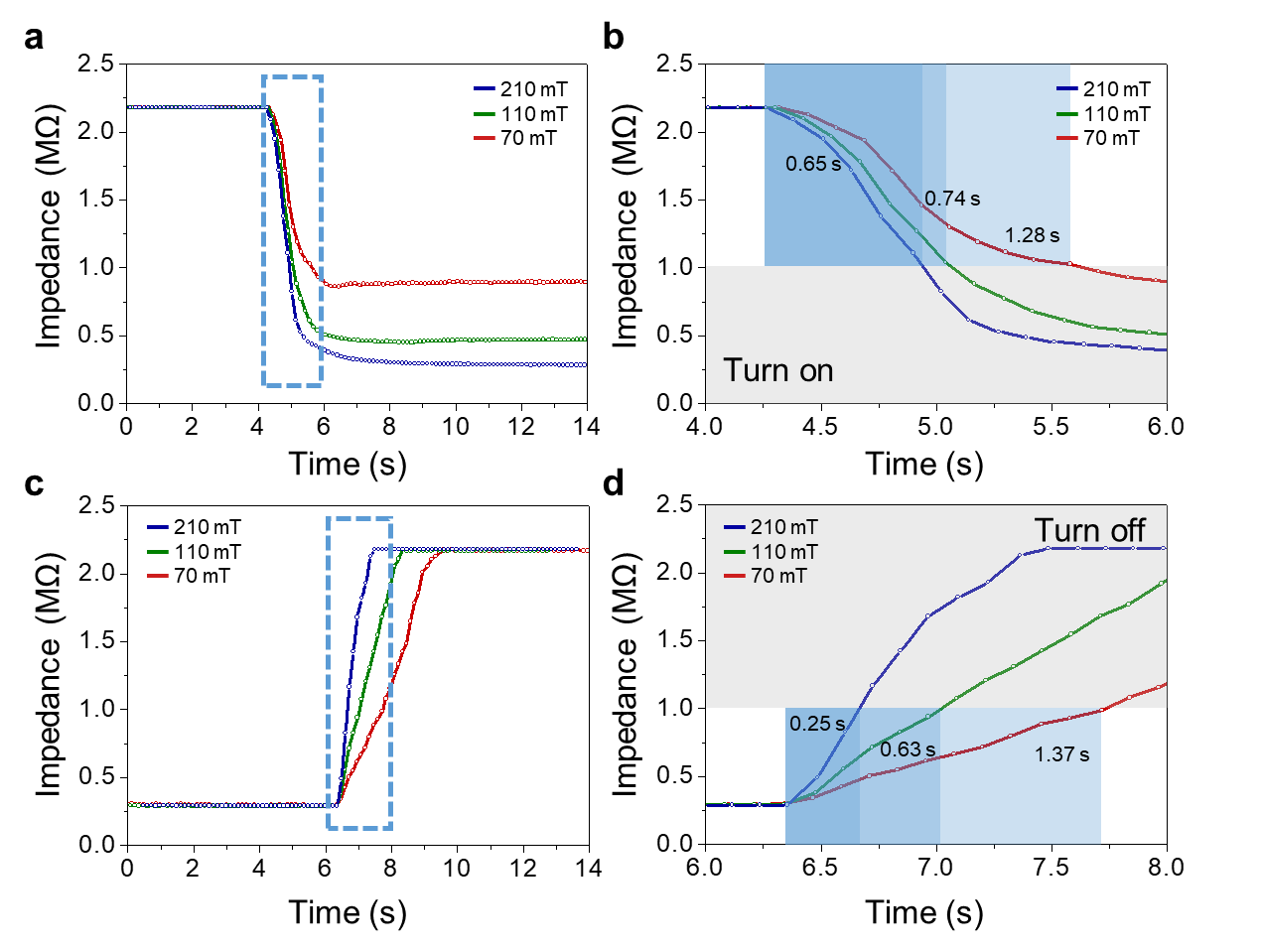


**Supplementary Figure 24.** Response time of the NV-MED in the impedance change with various magnetic fields. **a**. Time-resolved writing response with impedance change of the NV-MED under different magnetic fields of 70, 110, and 210 mT. **b**. Result of expanded writing response in (a). Writing time to reach turn-on (under ~1 MΩ) with different magnetic fields of 70, 110, and 210 mT. **c**. Time-resolved erase response with impedance change of the NV-MED under different magnetic fields of 70, 110, and 210 mT. **d**. Result of expanded writing response in (c). Erasing time to reach turn-off (above ~1 MΩ) with different magnetic fields of 70, 110, and 210 mT. The results of the impedance analysis are confirmed to be consistent with the actual turn-on/off response recorded by the optical acquisition system.


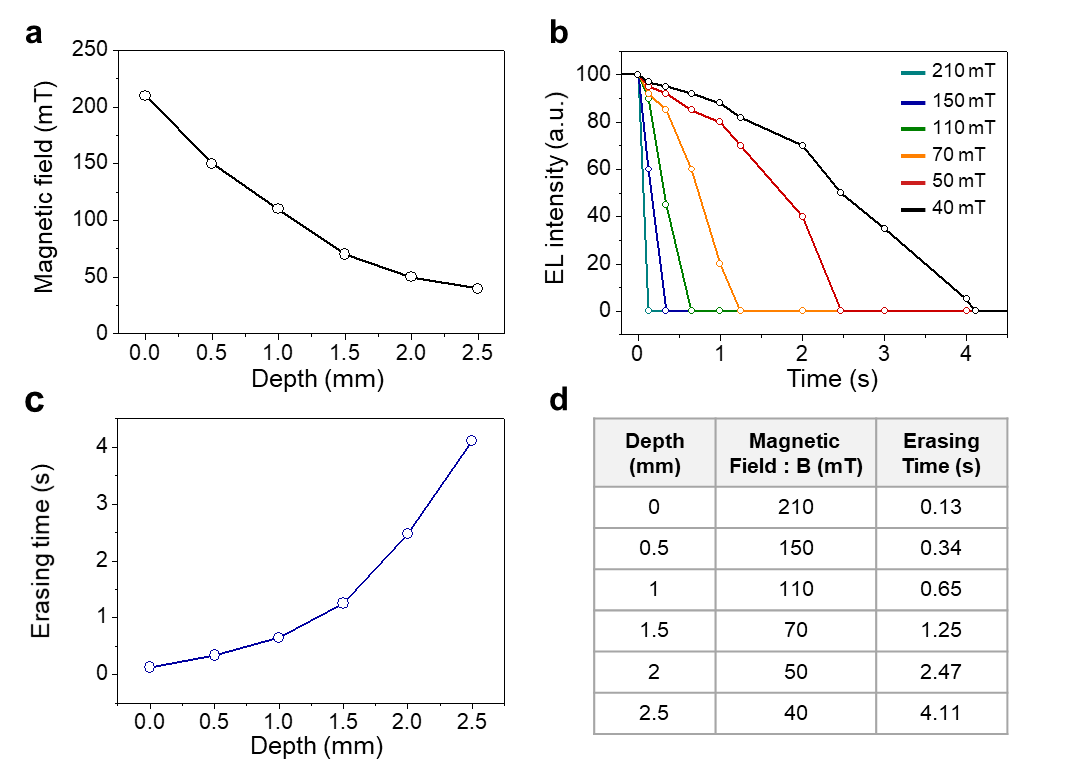


**Supplementary Figure 25.** Response time of the NV-MED in the EL change with various magnetic fields. **a**. The effective magnetic field as a function of probe depth. **b**. EL intensity decay of the NV-MED with time upon erasing with a given magnetic field. **c**. A plot of erasing time required to completely turn off EL as a function of probe depth. **d**. Table of the summary of experimental results shown in a, b and c.


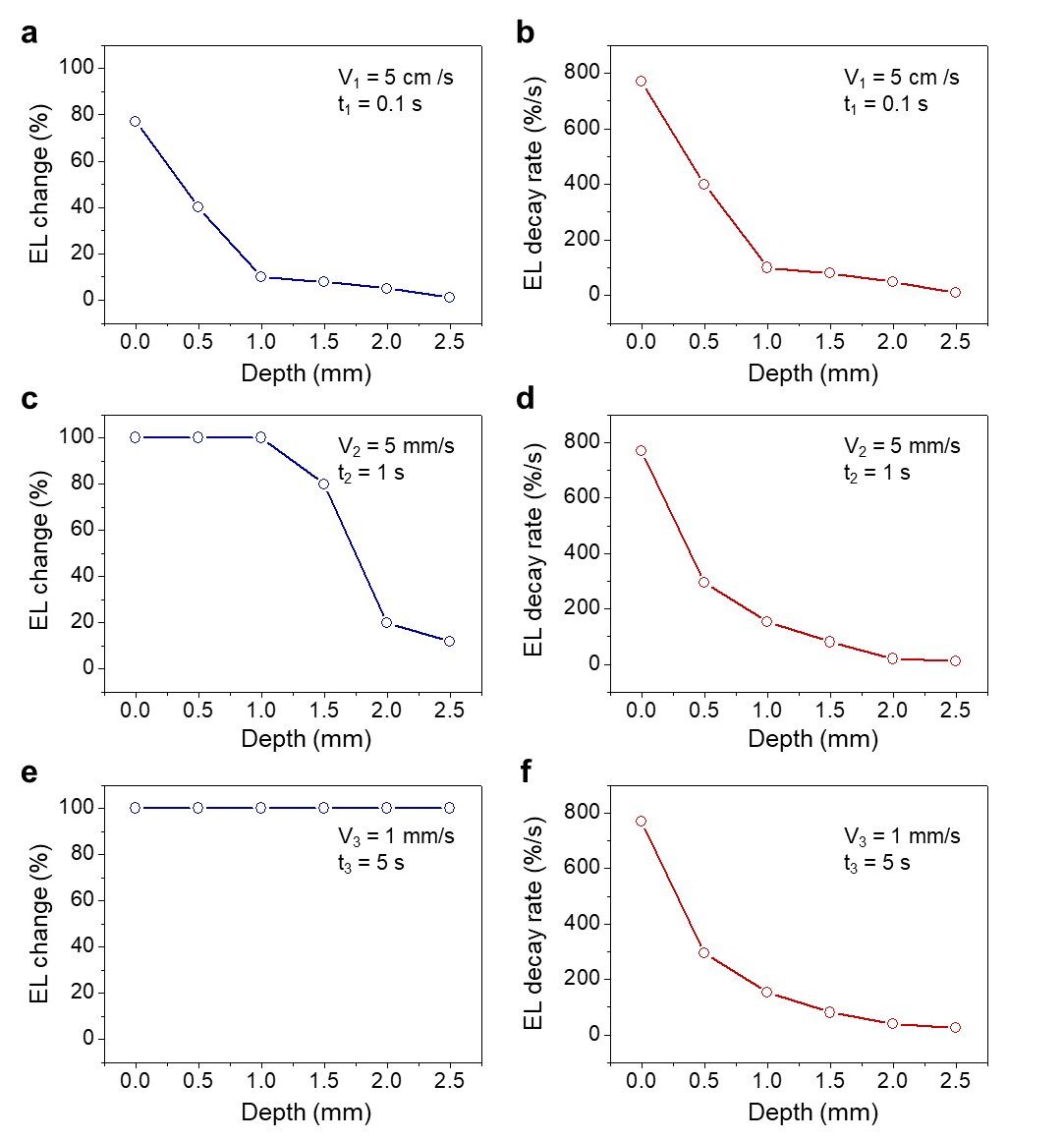


**Supplementary Figure 26.** Characteristics of EL of the NV-MED with various magnetic probe speed. EL intensity changes as a function of depth with the probe speed of (**a**) 5 cm/s, (**c**) 5 mm/s, (**e**) 1 mm/s. EL decay rates as a function of depth with the probe speed of (**b**) 5 cm/s, (**d**) 5 mm/s, (**f**) 1 mm/s. A constant probe speed is practically applied for reliable 3D motion tracking based on our NV-MEDs. In our system, the highest speed employed for the measurement was approximately 5 cm/sec below which the 3D tracking was appropriately performed. All similar plots of EL decay rate as a function of vertical depth were obtained, regardless of the probe speeds, while the plots of luminance change as a function of vertical depth were not consistent with the probe speeds, making the characterization with EL decay rate more reliable.


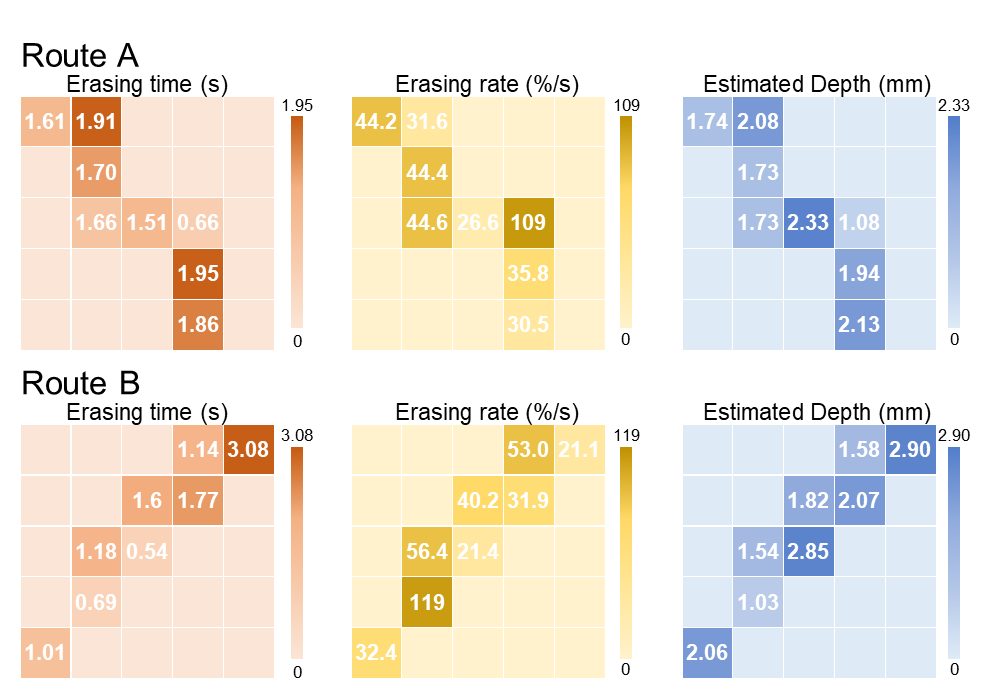


**Supplementary Figure 27.** Analysis of routes A and B through the optical brightness variation. Measured EL intensity of 5 × 5 NV-MED array with optical acquisition system. Extracted value of the EL erasing time, erasing rate, and depth of the magnetic surgical probe.

**
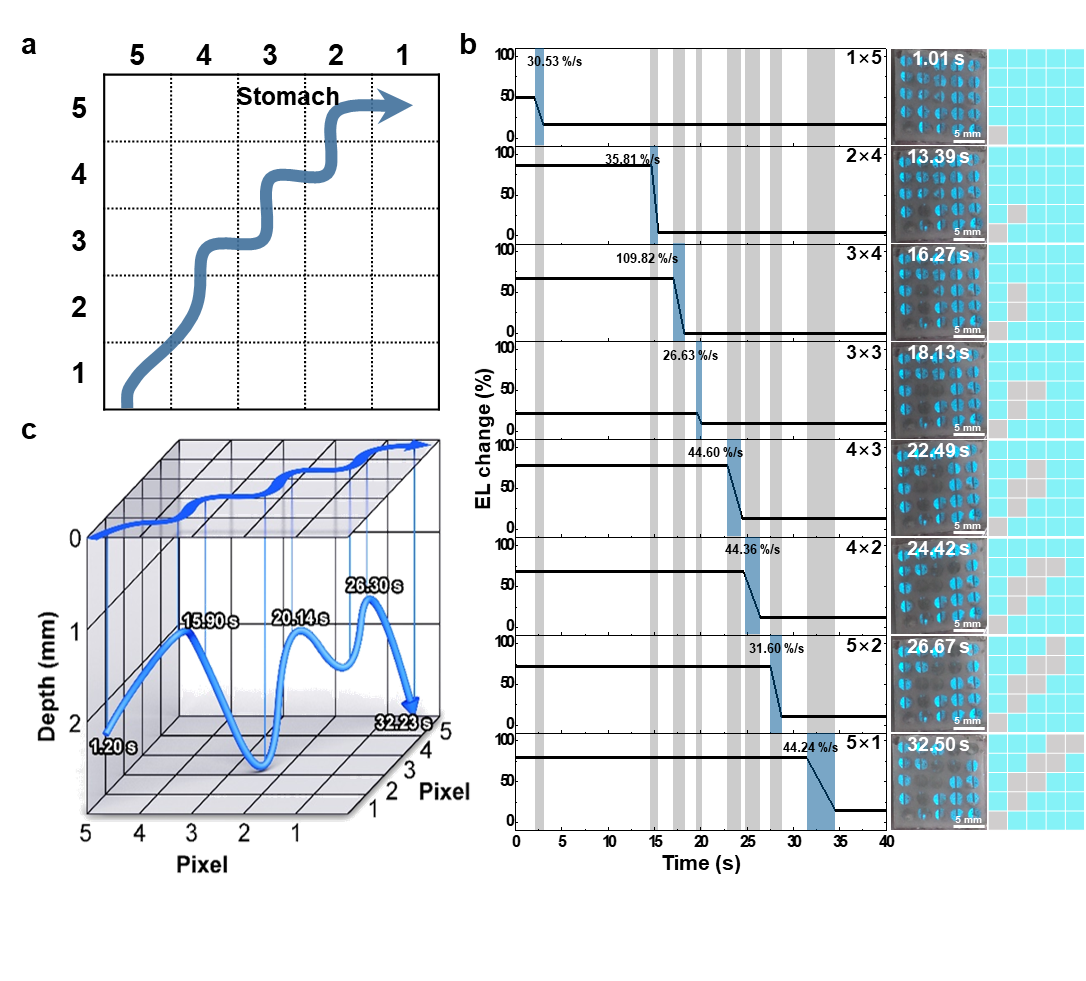
**

**Supplementary Figure 28.** Analysis of the depth of route B through the optical brightness variation. **a**. Estimated routes for stomach surgery with the grid. **b**. EL change in the pixels of NV-MED arrays as a function of time upon moving the magnetic probe to the target stomach. Images of the NV-MED arrays mounted on a rat captured during the movement. The time for each step is shown in the photograph (scale bars: 5 mm). **c**. Variation in the depth of the magnetic probe as a function of erasing rate. **h**. Three-dimensional plots of the route to the stomach with depth of the magnetic probe showing the actual path of the probe.


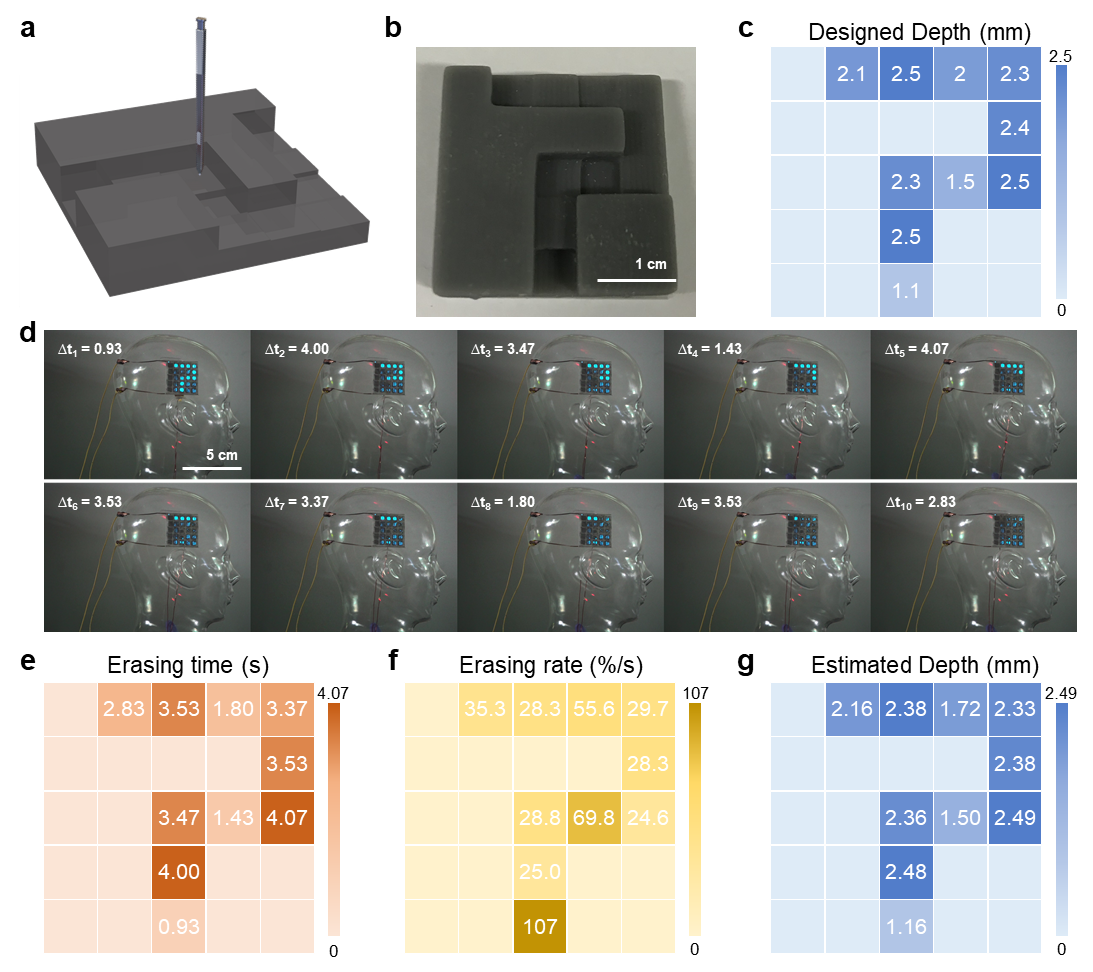


**Supplementary Figure 29.** *In-vitro* 3D motion tracking NV-MED display. **a**. Schematic of a 3D printed tracking mould. **b**. A photograph of a 3D printed tracking mould mounted on the NV-MED. **c**. Designed depth profile of 3D printed mould recorded on 5 × 5 NV-MED arrays. **d**. Captured photographs of NV-MED arrays mounted on a transparent human skull dummy upon moving the magnetic probe along the route of the mould. The time for each step is shown in the photograph (scale bars: 5 cm). **e**. Erasing time of NV-MED measured from each pixel array. **f**. Erasing rate of NV-MED calculated from erasing time of each pixel array. **g**. Estimated depth profile of 3D motion tracking of magnetic probe. The results show almost similar estimated depth value to the designed depth.


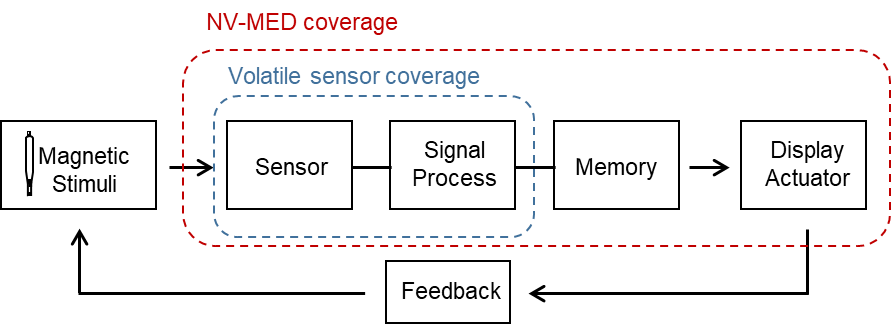


**Supplementary Figure 30.** Schematics of user-interactive 3D motion tracking display system. An external stimuli (magnetic) and Sensor-Signal Process-Memory-Display system must be comprehensively built. While volatile sensors can cover a part of the system, our NV-MED with non-volatile characteristics covers the entire sensing display system at a single device level.


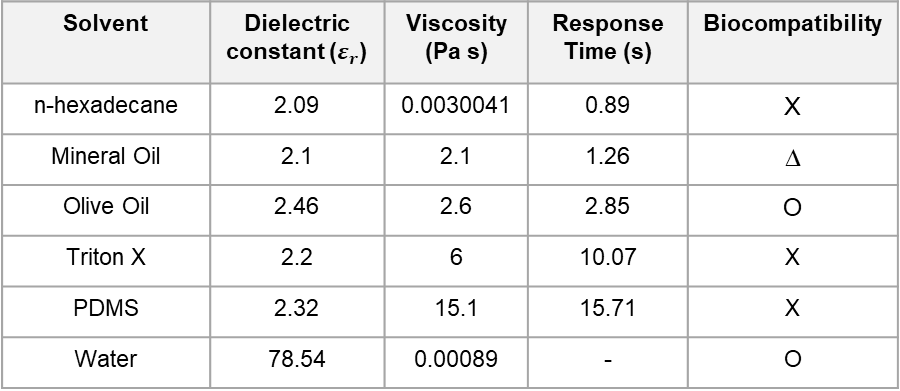


**Supplementary Table 1.** Characteristics of the various solvents suitable for NV-MEDs.
